# Supplementary material for: Carriage rates and antimicrobial sensitivity of pneumococci in the upper respiratory tract of children less than ten years old, in a north Indian rural community
Source: PLoS One. 2021 Feb 4;16(2):e0246522. doi: 10.1371/journal.pone.0246522 (PMC7861412; doi:10.1371/journal.pone.0246522)
Supplement: S1 Table — (PDF) [file pone.0246522.s002.pdf]

S1 Table. Pneumococcal serotypes distribution among 220 isolates from children in rural northern India, 2012-2014.

Serotypes	No. of isolates (%)	Cumulative percentage
6B/C	19 (8.6)	8.6
19A	16 (7.2)	15.8
19F	15 (6.8)	22.6
23F	14 (6.4)	29.0
35A/B/C	14 (6.4)	35.4
15B	11 (5.0)	40.4
14	10 (4.5)	44.9
11A/C/D	7 (3.2)	48.1
34/47	7 (3.2)	51.3
9V	7 (3.2)	54.5
10A/B	6 (2.7)	57.2
22A	6 (2.7)	59.9
7F/A/B	6 (2.7)	62.6
18A/B	5 (2.2)	64.8
23A	5 (2.2)	67.0
9A/L	5 (2.2)	69.2
15C	4 (1.8)	71.0
23B	4 (1.8)	72.8
35F	4 (1.8)	74.6
6A	4 (1.8)	76.4
33B/C/D	4 (1.8)	78.2
4	3 (1.4)	79.6
16F	3 (1.4)	81.0
17F	3 (1.4)	82.4
33A/F	3 (1.4)	83.8
1	2 (0.9)	84.7
20	2 (0.9)	85.6
10C/F	2 (0.9)	86.5
13/28	2 (0.9)	87.4
15A	2 (0.9)	88.3
18C/F	2 (0.9)	89.2
21/39	2 (0.9)	90.1
22F	2 (0.9)	91.0
5	1 (0.5)	91.5
29	1 (0.5)	92.0
36	1 (0.5)	92.5
48	1 (0.5)	93.0
11B/F	1 (0.5)	93.5
17A	1 (0.5)	94.0
19B	1 (0.5)	94.5
24B	1 (0.5)	95.0
9N	1 (0.5)	95.5
**Non-typeable**	10 (4.5)	100.0
