# Supplementary material for: Carriage rates and antimicrobial sensitivity of pneumococci in the upper respiratory tract of children less than ten years old, in a north Indian rural community
Source: PLoS One. 2021 Feb 4;16(2):e0246522. doi: 10.1371/journal.pone.0246522 (PMC7861412; doi:10.1371/journal.pone.0246522)
Supplement: S2 Table — AURI: Acute upper respiratory infection, ALRI: Acute lower respiratory infection, RSV: Respiratory syncytial virus, HRV: Human rhinovirus, HMPV: Human metapneumovirus, PIV: Parainfluenza virus, Hib: Haemophilus influenzae type b. (PDF) [file pone.0246522.s003.pdf]

S2 Table. Multi-variate analysis of factors associated with detection of pneumococcus in the upper respiratory specimens collected from children aged <10 years in rural north India.

Factors	Adjusted odds ratio	95% CI	p-value
**Age group**			
0 to <1 year	**18.4**	**(10.6-31.8)**	**0.000**
1 to <2 years	1.6	(0.8-3.2)	0.168
2 to <5 years	0.67	(0.3-1.4)	0.268
5 to <10 years	(Ref)		
**Sex**			
Male	1.1	(0.8-1.5)	0.648
Female	(Ref)		
**Type of ARI**			
AURI	**2.6**	**(1.5-4.4)**	**0.000**
ALRI	**2.0**	**(1.1-3.5)**	**0.017**
Asymptomatic control	(Ref)		
**Type of virus co-detected**			
Influenza	1.3	(0.4-4.0)	0.655
RSV	0.7	(0.3-1.9)	0.53
HRV	**2.2**	**(1.3-4.0)**	**0.006**
HMPV	0.4	(0.0-2.8)	0.332
PIV	0.6	(0.2-2.3)	0.488
Hib	**3.0**	**(1.4-6.2)**	**0.003**
**Months**			
January	(Ref)		
February	1.2	(0.6-2.5)	0.58
March	0.5	(0.2-1.1)	0.089
April	0.8	(0.4-1.6)	0.459
May	1	(0.5-2.0)	0.938
June	1.4	(0.7-2.8)	0.378
July	1	(0.5-2.1)	0.956
August	0.8	(0.3-1.7)	0.535
September	0.3	(0.1-0.7)	0.009
October	0.8	(0.3-1.8)	0.576
November	0.9	(0.3-2.6)	0.881
December	0.5	(0.2-1.4)	0.166
