# Supplementary material for: Carriage rates and antimicrobial sensitivity of pneumococci in the upper respiratory tract of children less than ten years old, in a north Indian rural community
Source: PLoS One. 2021 Feb 4;16(2):e0246522. doi: 10.1371/journal.pone.0246522 (PMC7861412; doi:10.1371/journal.pone.0246522)
Supplement: Attachment — Submitted filename: Response to Reviewers.rtf [file pone.0246522.s004.rtf]

Reviewers' comments:

Reviewer's Responses to Questions
Comments to the Author

1. Is the manuscript technically sound, and do the data support the conclusions?

The manuscript must describe a technically sound piece of scientific research with data that supports the conclusions. Experiments must have been conducted rigorously, with appropriate controls, replication, and sample sizes. The conclusions must be drawn appropriately based on the data presented.
Reviewer #1: Yes

2. Has the statistical analysis been performed appropriately and rigorously?
Reviewer #1: Yes

3. Have the authors made all data underlying the findings in their manuscript fully available?

The PLOS Data policy requires authors to make all data underlying the findings described in their manuscript fully available without restriction, with rare exception (please refer to the Data Availability Statement in the manuscript PDF file). The data should be provided as part of the manuscript or its supporting information, or deposited to a public repository. For example, in addition to summary statistics, the data points behind means, medians and variance measures should be available. If there are restrictions on publicly sharing data—e.g. participant privacy or use of data from a third party—those must be specified.
Reviewer #1: Yes

4. Is the manuscript presented in an intelligible fashion and written in standard English?

PLOS ONE does not copyedit accepted manuscripts, so the language in submitted articles must be clear, correct, and unambiguous. Any typographical or grammatical errors should be corrected at revision, so please note any specific errors here.
Reviewer #1: Yes

5. Review Comments to the Author

Please use the space provided to explain your answers to the questions above. You may also include additional comments for the author, including concerns about dual publication, research ethics, or publication ethics. (Please upload your review as an attachment if it exceeds 20,000 characters)
Reviewer #1: The title: Carriage rates and antimicrobial sensitivity of pneumococci in the upper respiratory tract of children less than ten years old, in a north Indian rural community
This topic is an important topic indicating pneumococcal resistance in children less than ten years with high risk to moderate risk of pneumococcal infections and mostly with high resistance rates for rural areas. The manuscript should have the following points for corrections
reviewer points:
1. Follow the author instructions regarding abstract writing, it should be in one paragraph.
Author: Correction changes, according to PloS One guidelines.
2. Line 28: anti-microbial, no need for the dash
Author: Correction changes.
3. again in the abstract methods, it is stated that you took only nasal swabs from children less than one year, but the rest children both nasal and throat, but in the results (nasal/throat for both), please explain.
Author: We took only nasal swabs from children less than one year old and throat swabs were taken from the rest children for bacterial detection, which are collectively representing in results either nasal or throat swabs, now corrections are changes in both abstract and results as well.
4. lines 34, 35 and 36: (with 6B/C, 19A, 19F, 23F, 35A/B/C, 15B, 14 and 11A/C/D
35 accounting for 50%). You should indicate the exact percentage of each serotype from the highest to the lowest, showing the importance of each and give the reader an idea about the coverage of the pneumococcal conjugate vaccines, and this may also indicate or show the importance of the future PCVs PCV15 or PCV20.
Author: Correction changes in abstract, exact percentage of each serotype from highest to lowest order included.
5. Line 37 in the abstract, it says less than 10% penicillin intermediate resistance: Please indicate the exact resistance rates and also high grade resistance for the penicillin.
Author: Out of 220 pneumococcal isolates, 22 (10%) were penicillin intermediate resistance with MIC ranges (0.125 to 1.5) of oral penicillin V, as follow the CLSI guidelines and no single isolates were having more than 2 MIC. So, in the current study pneumococcal isolates with high grade resistance for the penicillin were not found in this rural community. 
6. The introduction is mainly talking about India and is short, it needs extension and talk more internationally, especially in countries similar to India
Author: Correction changes, introduction extended and current trends of pneumococcal disease in neighbouring countries included in the manuscript now.   
7. line 66, same anti-microbial, no need for the dash
Author: Changes done.
8. The study is about Low and Upper RI, were there any pneumonia cases for the children diagnosed?
Author: There was no any pneumonia case diagnosed; just followed IMNCI and IMAI guidelines through appearance of physical respiratory disease sign and symptoms to consider children in either Lower or Upper RI group.
9. In line 79. change the sentence starting with we, since it is not scientifically correct
Author: Changes done accordingly.
10. lines 91-93: Is there any importance of including this statement?
Author: These lines just describe about asymptomatic children, which has included age matched similar number of ALRI.
11. Line 103: (for testing for), here English revision
Author: Correction changes.
12. Line 104: be sure to make all CO2 written the same in the manuscript (the number 2 should be low script)
Author: Correction changes.
13. make sure there are no double spaces between words in all of the manuscript, look at line 119.
Author: Changes done.
14. line 127, the reference was put after the point (wrong)
Author: Correction changes.
15. lines 124-130: You have mentioned in the abstract penicillin intermediate resistance and even in line 199 about penicillin resistance, but penicillin was not mentioned in the methods in this paragraph
Author: Correction changes in methods accordingly.
16. line 132: What is s/he?
Author: s/he represent female or male child, had included in new case of ARI and depends on onset of disease either AURI or ALRI.
17. I do not encourage you to write we analyzed, we considered, we used, we also detected, We identified...etc. This is not preferable in the manuscripts
Author: Yes you are right, correction changes accordingly.
18. lines 153-154: This included 162 specimens collected within 14 days of last specimen collection from the same child. Please clarify 162 samples from the same child?
Author: These lines meant to say that some children were sampled multiple times within 14 days and there were 162 such specimens. We have edited this sentence in the manuscript to state “There were 162 specimens from 131 children which were collected within 14 days of last sample collection”.
19. Indicate the serotypes in the figure with PCV serotypes or not
Author: Yes, it's mentioned in Fig.2 indicating serotype with individual PCV (23, 13, 10) serotypes
